# Examining the impact of familiarity on faucet usability for older adults with dementia

**DOI:** 10.1186/1471-2318-13-63

**Published:** 2013-06-20

**Authors:** Jennifer Boger, Tammy Craig, Alex Mihailidis

**Affiliations:** 1 Toronto Rehabilitation Institute, University of Toronto, 160-500 University Ave, Toronto, ON M5G1V7, Canada; 2 University of Toronto, Toronto, ON, Canada

**Keywords:** Familiarity, Product usability, Older adults, Dementia, Enabling independence, ADL completion, Human factors, Product design

## Abstract

**Background:**

Changes in cognition caused by dementia can significantly alter how a person perceives familiarity, impacting the recognition and usability of everyday products. A person who is unable to use products cannot autonomously complete associated activities, resulting in increased dependence on a caregiver and potential move to assisted living facilities. The research presented in this paper hypothesised that products that are more familiar will result in better usability for older adults with dementia. Better product usability could, in turn, potentially support independence and autonomy.

**Methods:**

This research investigated the impact of familiarity on the use of five faucet designs during 1309 handwashing trials by 27 older adults, who ranged from cognitively intact to the advanced (severe) stages of dementia. Human factors methods were used to collect empirical and self-reported data to gauge faucets’ usability. Participants’ data were grouped according to cognition (i.e., no/mild, moderate, or severe dementia). Logistic regression, ranking by odds, and Wald tests of odds ratios were used to compare performance of the three groups on the different faucets. Qualitative data were used in the interpretation of quantitative results.

**Results:**

Results indicated that more familiar faucets correlated with lower levels of assistance from a caregiver, fewer operational errors, and greater levels of operator satisfaction. Aspects such as the ability to control water temperature and flow as well as pleasing aesthetics appeared to positively impact participants’ acceptance of a faucet. The dual lever design achieved the best overall usability.

**Conclusions:**

While work must be done to expand these findings to other products and tasks, this research provides evidence that familiarity plays a substantial role in product usability for older adults that appears to become more influential as dementia progresses. The methods used in this research could be adapted to analyse usability for other products by older adults with dementia.

## Background

Longer life spans and lower birth rates are causing the global average age to increase. The number of people over the age of 65 is expected to almost triple from 523 million in 2010 to just under 1.5 billion by 2050 [1,2]. Reflecting trends in aging, the global number of people with dementia is predicted to increase from 24.3 million people in 2001 to 81.1 million in 2040 [3,4]. The changes in cognition caused by dementia makes the completion of activities of daily living (ADL), such as toileting and handwashing, increasingly difficult as symptoms become more severe. A person’s ability to complete ADL is not only necessary for physical well-being, but is central to one’s independence, pride, and dignity [5,6]. An inability to complete ADL often results in a dependence on caregivers, thus increasing caregiver burden and care costs [7-9]. The associated social and economic costs (both direct and indirect) for caring for older adults, particularly those with dementia, is expected to be enormous. In 2010, the global cost of supporting people with dementia was estimated to be US$604 billion, an amount equivalent to 1% of the world’s GDP [10]. There is an acute need not only for an increase in assisted-living housing and restructuring of medical services, but also for the implementation of everyday support for this population [11,12].

Morbidities that occur naturally with aging, such as reduced vision, dexterity, hearing, and speed, can make many products difficult or impossible to use. Cognitive impairments caused by dementia result in additional (often quite substantial) difficulties when trying to use even simple everyday products, resulting in a greater reliance on caregivers. Increasing products’ usability has the potential to inherently support peoples’ independence and autonomy while simultaneously reducing caregiver burden. Being familiar with a product and knowing how to use it plays a crucial role in how usable a product is. Kaplan & Kaplan [13] propose that familiarity describes the relationship between an individual and something the individual has considerable experience with. Sufficient experience leads to the development of an internal model, or stereotype, about how one expects something to work. Well-designed products often achieve good usability by leveraging familiarity through the incorporation of stereotypes and peoples’ expectations of how something should work, even if they have not used the specific product before [14].

Dementias such as Alzheimer’s disease impair memory functioning, with short-term and explicit (declarative or conscious) memory usually considerably more affected than long-term and implicit (habitual or unconscious) memory [15,16]. In other words, older, well-rehearsed memories generally remain relatively accessible while newer experiences do not. As people with dementia’s long-term memory is relatively spared, it follows that the history of previous exposures to something will have a greater impact on familiarity than how recent the exposure was. Son, Therrien, and Whall [17] support this theory, proposing that the more things take advantage of people with dementia’s relatively preserved implicit memory, the greater their impact will be on maintaining or enhancing functional abilities. Publications regarding environmental design, such as [17-23], incorporate this notion, recommending environments that feel familiar, are non-threatening, have an intuitive design, and allow for personalisation with familiar objects. Research has indicated that appropriately designed environments can compensate for decreased cognitive abilities, resulting in increased autonomy and positively impacting behaviours by supporting the care, well-being, and functionality of people with dementia [22,23]. Research regarding devices for people with dementia recommends similar approaches, advocating solutions that complement the abilities of the intended user population [24-29]. For example, through discussions, focus groups, and a questionnaire, Orpwood et al. [24] derived user-needs criteria for smart home technologies for supporting dementia. Recommendations included creating interfaces and devices that look familiar and operate in a simple, stereotypical way to encourage a person with dementia to interact with the device. It is understandable that people with dementia would feel more comfortable and confident interacting with environments and devices they implicitly recognise and understand.

While environmental and assistive technology design are examples of areas that have incorporated or specifically targeted older adults with dementia, there is a lack of research regarding how the design of everyday products impacts product usability for this population. The research cited above reason that if something looks familiar to an older adult with dementia, then she or he will be much more likely to understand its meaning and therefore be able to use it; the environment or device will be more usable for them. Better usability through implicit recognition of everyday products could result in increased ADL completion, improving independence, well-being, and feelings of self-esteem while simultaneously reducing caregiver interventions and burden. Conversely, incomprehension or non-recognition will impede product use and hamper autonomous completion of associated tasks.

This paper presents research that methodically investigated the impact of familiarity on product use by investigating the use of five different bathroom faucets by older adults with varying degrees of cognition to complete the activity of handwashing. Being able to use a faucet is critical to accomplishing many of the activities necessary for independence (e.g., toileting, handwashing, preparing a meal). Handwashing was selected as a representative ADL as it is an activity people do many times a day, is relatively quick, elicits full use of a faucet’s functions (i.e., water flow and temperature adjustment), is usually challenging for older adults with dementia to complete independently, and is familiar to the authors through previous research [30-33].

The primary hypothesis for this research was that lifetime exposure (familiarity) with a faucet design has a greater impact on how usable the product is for older adults with dementia than the products’ usability (as defined by a human factors approach), with more familiar products being more usable. Furthermore, it was hypothesised that the impact familiarity has on usability becomes more pronounced as dementia severity increases.

**Figure 1:**
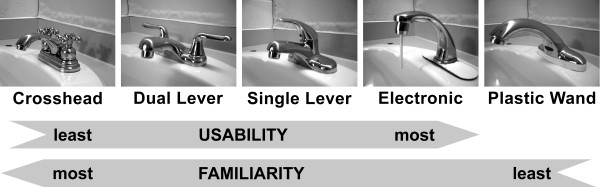
**The five faucet designs used in this study.** Faucets were ordered from least usable (left) to most usable (right) based on a human factors approach and most familiar (left) to least familiar (right) based on average years of exposure and commercial availability. The plastic wand has not yet had a formal usability study, therefore could not be rated for usability. While familiarity plays a role in usability, the faucets were ordered independently for both usability and familiarity.

As presented in Figure 1 and discussed in detail in the following sections, faucet usability can be at odds with faucet familiarity. However, if familiarity plays a crucial role in product recognition and use by older adults with dementia, then familiarity should dictate successful product use, even with products that have sub-optimal usability from a human factors point of view. Therefore, it was theorised that if more familiar faucets corresponded to higher levels of correct and unassisted faucet use, this likely indicated that familiarity plays an essential, and possibly critical, role in how usable a faucet is for older adults with dementia. The following sections present the study’s methodology and results, followed by a discussion of the faucets’ performance before concluding with a summary of the studies’ findings and directions for future research.

## Methods

### Faucet selection

The five faucets used in this study were (Figure 1): crosshead (Danze Sheridan, Model D302165), dual lever (American Standard Cadet, Model 8125), single lever (Cadet, Model 6211), infrared (Delta Synergy 4", Model 591T1250), and a novel plastic wand (Automatic Faucet Control obtained from the Alzheimer’s Store, Ageless Design Company). With the exception of the plastic wand, all faucets were “off the shelf” models.

### Faucet familiarity

Ideally, faucet familiarity would be determined by identifying all the faucet designs one has been exposed to over a lifetime, particularity the designs used at home and other familiar environments. However, this is not a practical approach as most people (particularly those with dementia) cannot explicitly or reliably recall these details. Therefore, this research considered a faucet to be more familiar the longer it had been in the consumer market. A search of grey literature, such as product advertisements in home furnishing magazines dating back to the 1920s, was used to establish when different faucets became available to the public and was used to create the familiarity ordering in Figure 1. The crosshead design was identified as the most familiar as it has been available for the longest period of time and has achieved widespread use. The infrared was considered to be the least familiar of the four commonly-encountered faucet designs as it is the most recent faucet to appear in the marketplace and is primarily found in shared (public) washrooms. The novel plastic wand design was considered to be the least familiar overall.

### Faucet usability

In a human factors context the term usability reflects how well a person is able to achieve an intended goal or goals when interacting with an interface or item [34-36]. Usability can be defined by three aspects: effectiveness, efficiency, and satisfaction [34,36,37]. As defined by ISO 9241–11 [36], effectiveness is the accuracy and completeness with which users achieve goals, efficiency the resources expended in relation to the accuracy and completeness with which users achieve goals, and satisfaction the comfort and acceptability of use. These concepts were used to create the faucet usability ordering presented in Figure 1. The crosshead faucet’s actuators (i.e., handles) makes it the most difficult design to use; the operator must grasp and rotate the actuators to operate the faucet and the rotational operation of the actuators makes it easier to make an error. The simpler push/pull action of the dual lever’s actuators make it easier to operate and make it a more intuitive design. The single lever faucet requires even less effort, needing only a light touch with any part of a hand or arm to achieve simultaneous temperature and flow control. The infrared faucet requires the least amount of effort as it turns on when a hand (or other object) is placed in front of the infrared sensor. Moreover, the water shuts off automatically and the water temperature cannot be adjusted, eliminating these steps. With the plastic wand, the operator must move the wand away from centre in any direction (e.g., push, pull, or sideways movement) to make the water flow. Releasing the wand causes it to return to a centre position and the water flow stops. To the authors’ knowledge, there have been no usability studies for the plastic wand, therefore its usability could not be rated.

### Measuring faucet usability by older adults with dementia

#### *Effectiveness: Types and number of errors made by the participant*

Effectiveness can be measured by the number and types of errors that are made by the participant. For this study, faucet operation errors were classified as five types, listed in Table 1.

**Table 1:** Types of faucet operation errors

**Error type**	**Error description**
No error	The participant used the faucet without committing an error
Wrong location	The participant interacted with the faucet, but did not use the actuator (e.g., the participant attempted to turn on the water using the spigot, rather than the actuator).
Wrong operation	The participant interacted with the faucet’s actuator, but did not use it correctly (e.g., pushing down on a knob instead of rotating it)
Wrong outcome	The participant interacted with the actuator correctly, but did not achieve the desired outcome (e.g., the participant wanted to increase water flow, but turned the knob in the wrong direction, unintentionally turning the water off)
No attempt	The participant did not attempt to use the faucet

### Efficiency: Types and amount of assistance given to participant

Efficiency can be measured in terms of effort and time. If using one product requires less effort compared to another product, it can be said that the first product has improved the efficiency of the task. If a task requires caregiver intervention, then it likely requires too much effort for the person with dementia and the caregiver, too, must expend effort to complete the task. Moreover, the amount of caregiver assistance is an important consideration as unassisted product use is crucial to independent ADL completion. Thus, the amount of caregiver assistance required (or lack thereof) can be used as a measure of the effort involved. Caregiver assistance was classified as seven types, which are listed in Table 2.

**Table 2:** Types of caregiver assistance

**Assistance type**	**Assistance description**
No assistance	The participant completed the step independently
Verbal 1	A simple verbal cue was given by the caregiver to orient the participant to the step s/he was attempting (e.g., “Can you turn on the water?”)
Verbal 2	A verbal prompt was given by the caregiver specifying how to accomplish the step (e.g., “Try pulling the lever toward you”)
Demonstrative 1	Caregiver pointed to or lightly touched the region of interest with accompanying verbal instruction (e.g., touching the handle of the faucet while saying “Try turning on the water”)
Demonstrative 2	Caregiver demonstrated how to complete the step with accompanying verbal instruction (e.g., turning the tap on then off to demonstrate how it works while saying “Try turning on the water like this”)
Hand-over-hand	Taking the participant’s hand into her own, the caregiver and participant completed the step together
Completed by caregiver	The caregiver completed the step

### Satisfaction: Participants’ opinions regarding required effort

Satisfaction with a product can be difficult to quantify, as this is a subjective aspect that is influenced by factors such as context and personal preferences. Most techniques used to gauge satisfaction (e.g., exit surveys, rating of features, preference ordering, etc.) cannot be used reliably with people with dementia. However, previous research has used Likert scale questions with older adults with cognitive impairments to elicit useful and valid self-reported information, such as level of pain [38] and quality of life [39-41]. Likert questions are also a commonly used technique to gauge product satisfaction in general [42].

The authors developed a single four-point Likert question to gauge participants’ self-perceived difficulty when using a faucet that was verbally administered in two stages. After using a faucet, the participant was asked, “Do you think this faucet is easy or difficult to use?” If the participant answered “easy” he or she was then asked, “Very easy or kind-of easy?” If the participant answered “difficult”, he or she was asked, “Very difficult or kind-of difficult?” Participants who had a cognitive impairment were asked the satisfaction question after every trial (for up to ten responses per faucet) while cognitively intact participants, whose answers were considered to be more reliable, were asked after the first, fifth, and tenth trial to avoid frustrating the participant (for up to three responses per faucet). Participants were encouraged to share their opinions regarding the faucet throughout and after trials, including whether they would like to use the faucet in their home.

### Participants

Study inclusion criteria were: be 65 years of age or older; understand English; have no history of aggression; be able to see and hear (both could be with correction), and; have no diagnosis of Parkinson’s disease or a severe motor impairment. Informed consent was obtained from the participant or from his or her substitute decision maker as appropriate before entry into the study.

The Mini-Mental State Examination (MMSE) was used to estimate participants’ cognition. The MMSE is a standardised tool developed to gauge adult cognitive abilities by the interpretation of a subject’s score. Guided by research by [43,44], participants were assigned to one of four groups: cognitively intact/aware (MMSE of 30 to 25); mildly impaired (MMSE of 24 to 20); moderately impaired (MMSE of 19 to 10); and severely impaired (MMSE of 9 to 0). The MMSE was administered at the beginning, middle, and end of the study and averaged to give a participant’s overall score.

### Study procedure

Trials were conducted in a designated washroom at a long-term care facility in Toronto, Canada. Videos of the sink area were recorded to enable post-trial analysis. All data was collected, stored, and analysed according to a protocol that was approved research ethics (IRB, University at Buffalo and REB, University of Toronto).

Two researchers were present for all trials; one researcher, who had experience in geriatrics, acted as the caregiver (hereafter referred to as ‘the RA’) and one researcher operated study-related equipment. For safety and study uniformity, the RA sat the participant in a wheelchair at the beginning of each trial. The RA positioned the participant at the sink and asked him or her to wash his or her hands. The RA provided assistance to the participant only if and when the RA deemed it necessary (e.g., the participant was off-task, requested assistance, appeared anxious, etc.). For each handwashing step (i.e., turning on the water, getting soap, etc.), the RA began with the lowest possible type of assistance (see Section ‘Efficiency: Types and amount of assistance given to participant’) and only provided the next type if the participant required greater assistance. Scripted verbal prompts were used to ensure that assistance was as uniform as possible. Upon completion of the trial, the participant was asked the questions regarding satisfaction outlined in Section ‘Satisfaction: Participants’ opinions regarding required effort’.

A counterbalanced study design is the preferred method for assessing usability because it employs a balanced presentation of the conditions being tested to minimise possible confounding factors [42]. The sample size for a complete counterbalanced study is n!, where “n” represents the number of conditions being tested. As there were five faucets (conditions), this results in 5! = 120 subjects per group (level of cognition) being tested. As four groups were being tested (aware, mild dementia, moderate dementia, and severe dementia), this would result in a total of 480 participants. This was not a practical sample size for this stage of research, therefore a modified incomplete counterbalanced measures design was selected. The study aimed to recruit eight participants for each group, for a total of 32 participants. Each of the faucets was presented randomly to a participant for ten consecutive trials before the next (randomly selected) faucet type was introduced, for a total of up to 50 trials per participant. The presentation order of the faucets was unique for each participant to negate possible data trends caused by priming or fatigue. Participants completed one trial per day, excluding weekends.

### Data analysis

While participants completed the entire activity of handwashing, only the step of turning the water on was common to all faucet types. For instance, when using the infrared or plastic wand the operator cannot adjust water temperature or flow and cannot shut the water off as this happens automatically. As an analysis across the entire handwashing task would not be a balanced comparison of the faucet designs, only data pertaining to the water on step were analysed in detail and are presented in this paper.

While task time is a metric that is often used to gauge efficiency, it was not included in this study as it was not clear what it signified. Specifically, if a participant with dementia enjoyed using a faucet then they tended to take their time and interact more with it, however, longer task times were also seen for faucets that participants had trouble using. Additionally, it was felt that if a product enables someone from this population to complete a task independently, how long it takes to do so is relatively unimportant.

A researcher with extensive experience in scoring handwashing trials used the video footage to annotate the data. The rater scored the number and types of errors and assistance given in each trial and transcribed participants’ comments. To validate data reliability, a second rater scored three randomly selected trials for each subject. Inter-rater agreement between the primary and secondary raters was examined using Cohen’s kappa [45].

A visual analysis of the data was carried out to identify any learning effects across trials by comparing the mean amount and types of assistance required for trials 1–3, 4–6, and 7–10 for each group and faucet type. As participants from all groups were able to use all faucet types to some extent and as they were considered to be measurements of effectiveness and efficiency, statistical analyses were performed on two endpoints: 1) whether an error was committed and 2) whether assistance was required. These endpoints were summarized separately for each dementia group by faucet type.

Due to the small number of participants with mild dementia (n=3), analysis was conducted with the mild participants grouped with the aware participants, thus the remainder of this paper presents results from the aware/mild, moderate, and severe groups. As the data was correlated and clustered, logistic regression models were used to examine the relationship between faucet type and the two endpoints. Odds were obtained and used to rank the faucets for the three groups (i.e., “aware/mild”, “moderate”, and “severe”) for the likelihood of completely errorless and completely independent faucet use. Wald tests of odds ratios were used to test for equality of faucets. SAS v9.1 for Windows (SAS Institute Inc., Cary, NC, USA) was used for all statistical calculations. Descriptive statistics were used to analyse satisfaction data, which was captured through responses to the Likert question and comments made by the participants.

## Results

Twenty-seven (27) participants were recruited from the same long-term care facility in Toronto, Canada. Due to time and health-related complications, not all participants were able to complete 10 trials on each faucet type. Study demographics are presented in Table 3.

**Table 3:** Study demographics

**Group**	**Aware**	**Mild**	**Moderate**	**Severe**	***Total***
n (female)	9 (5)	3 (3)	9 (9)	6 (5)	*27* (*22*)
Average age (SD)	75.7 (8.9)	85.0 (3.6)	84.0 (8.0)	86.3 (9.5)	-
Average MMSE score (SD)	28.2 (1.5)	21.3 (0.9)	15.2 (2.3)	3.3 (2.9)	-
Average number of years in long-term care (SD)	3.0 (2.1)	1.0 (0.0)	2.8 (1.9)	2.5 (1.5)	-
Trials per faucet:					
Crosshead	85	28	89	57	*259*
Dual lever	87	29	90	60	*266*
Single lever	87	30	90	58	*265*
Infrared	80	30	88	60	*258*
Plastic wand	84	30	90	57	*261*
*Total*	*423*	*147*	*447*	*292*	*1309*

Cohen’s kappa for the two raters was calculated to be 0.90 for errors and 0.65 for assistance. As kappa values greater than 0.61 represent good agreement, the data compiled by the primary rater was considered to be a good representation of the trials [45].

**Figure 2:**
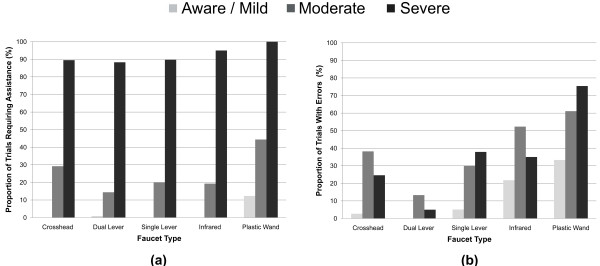
Proportion of the trials where: a) one or more errors were made by the participant when attempting to turn the water on and b) assistance was required by the participant to turn the water on.

**Figure 3:**
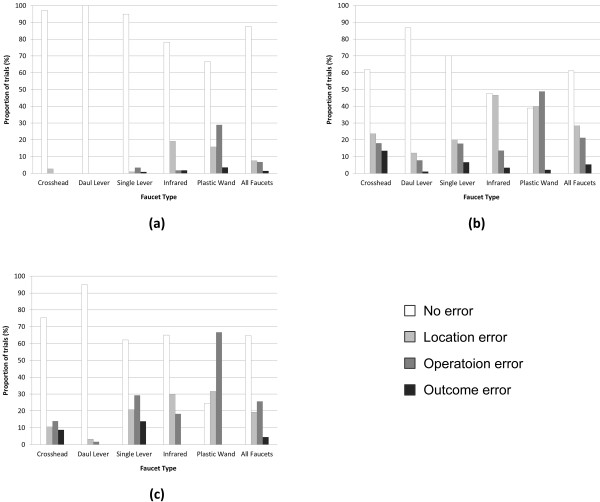
**Types of errors made for the (a) aware/mild, (b) moderate, and (c) severe levels of dementia groups.** Note that more than one type of error could be made in the same trial, therefore the sum of the errors for a faucet type can be more than 100% (e.g., the moderate group’s use of the plastic wand; 38.9% + 40% + 48.9% + 2.2% = 130%).

**Figure 4:**
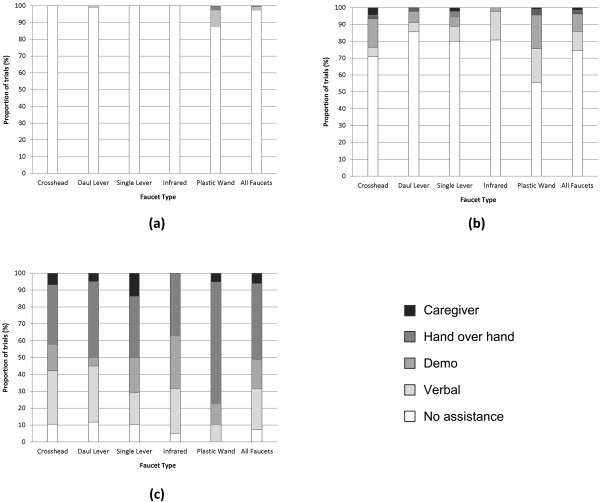
Highest type of assistance required during each trial when turning the water on for the (a) aware/mild, (b) moderate, and (c) severe levels of dementia groups.

The proportion of trials errors where were committed and assistance was given for turning on the water are shown in Figure 2. Types of errors and assistance were examined and are presented in Figures 3 and 4, respectively. Being able to turn the water on without committing an error or requiring assistance was considered to represent a faucet that promoted autonomy, therefore faucets were ranked by their calculated odds of a person being able to do so. These results are presented in Table 4. The fewest errors committed when using the more familiar dual lever, crosshead, and single lever designs (Figures 2a and 3 and Table 4) with the aware/mild group committing noticeably fewer errors when than the moderate and severe groups. Assistance (Figure 2b and 4) and odds rankings (Table 4) show that faucets generally require about the same amount and types of assistance for the same stage of dementia, with the dual lever and infrared performing slightly better.

**Table 4:** Faucets ranked by the odds of a person requiring any assistance and the odds of committing no errors when using the faucet to turn on the water for handwashing

**Group**	**Odds based rank**	**Required assistance**			**Committed no errors**		
		**Faucet**	**Odds**	P^*^<0.05	**Faucet**	**Odds**	P^*^<0.05
Aware/Mild^†,§^	1	Crosshead	-		Dual Lever	-	-
	2	Single Lever	-	-	Crosshead	36.67	I, PW
	3	Infrared	-	-	Single Lever	18.50	I, PW
	4	Dual Lever	0.01	PW	Infrared	3.58	C, SL
	5	Plastic Wand	0.14	DL	Plastic Wand	2.00	C, SL
Moderate	1	Dual Lever	0.17	C, PW	Dual Lever	6.50	C, SL, I, PW
	2	Infrared	0.24	C, PW	Single Lever	2.33	DL, I, PW
	3	Single Lever	0.25	PW	Crosshead	1.62	DL, PW
	4	Crosshead	0.41	DL, I, PW	Infrared	0.91	DL, SL
	5	Plastic Wand	0.80	C, DL, I, SL	Plastic Wand	0.64	C, DL, SL
Severe^‡^	1	Dual Lever	7.57	SL, I	Dual Lever	19.00	C, SL, I, PW
	2	Crosshead	8.50	I	Crosshead	3.07	DL, PW
	3	Single Lever	8.67	DL	Infrared	1.86	DL, PW
	4	Infrared	19.00	C, DL	Single Lever	1.64	DL, PW
	5	Plastic Wand	-	-	Plastic Wand	0.33	C, DL, SL, I

^*^ Wald Chi-sq P-values were calculated using pairwise comparisons run between faucet types within the same dementia group; C=crosshead, SL=Single Lever, DL=Dual Lever, I=Infrared, PW=Plastic Wand.

^†^ P-values could not be calculated for the crosshead, single lever, or infrared for the aware/mild group regarding assistance as these faucets required no assistance with these faucets.

^‡^ P-values could not be calculated for the plastic wand regarding assistance with the severe group as all participants required assistance with this faucet.

^§^ A pairwise comparison could not be done with the dual lever regarding errors with the aware/mild group as no participants committed an error using this faucet.

**Figure 5:**
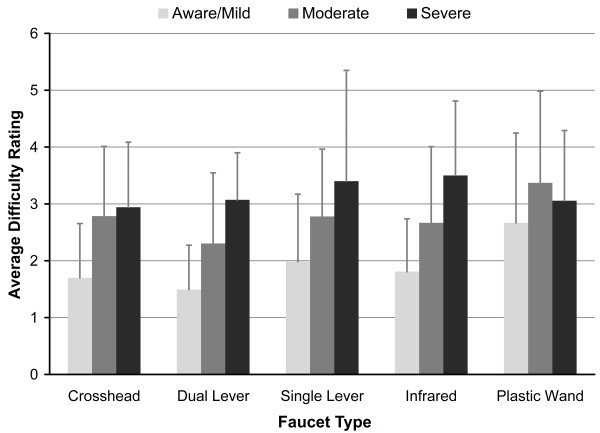
**Average self-reported level of difficulty in response to the question “Do you think this faucet is easy or difficult to use?”** Answers corresponded to a value on a four-point Likert scale where: 1 = very easy, 2 = easy, 3 = difficult, 4 = very difficult.

The average responses to the perceived difficulty question are shown in Figure 5. Only responses that matched Likert values were included (e.g., answers such as “I don’t find it difficult” and “No, I do not think it’s easy at all” were not included). Participants unequivocally answered the question 100% of the time it was posed to the aware group, 90.7% for the mild group, 82.2% for the moderate group, and 21.3% for the severe group (this rate increases to 42.6% when the three participants who never answered the question are removed). The decreasing response rate to the satisfaction question with increasing level of dementia reflects participants’ increasing inability to comprehend what was being asked as well as being less responsive in general. While some of the participants from the severe group gave opinions regarding difficulty, most had trouble with verbatim responses to the Likert question. As only unequivocal responses were considered, this contributed to the lower response rate for the severe group. While the standard deviations for all three groups are fairly large (Figure 5), they are also fairly similar. This suggests that participants from the different groups answered the perceived difficulty question with similar reliability. Comments made by the participants reflected that older adults, both with and without dementia, consider aesthetics to be an important aspect of a faucet and value the ability to control the water temperature and flow.

While the participants themselves recognised the value of different faucet types for different applications (e.g., “In public places this is good,” regarding the infrared faucet), they expressed a clear desire for control over the flow and temperature of the water. General comments made by participants indicated a shift from observations about the faucet itself to questions regarding how to complete the task as dementia progressed. For example, the aware/mild group mostly commented on aesthetics and personal preferences regarding operation modality. Comments from the moderate group showed signs of confusion, such as “It doesn’t like me sometimes; turning it on is difficult,” and “It wouldn’t be difficult once you got used to it,” while comments from severe group reflected incomprehension, such as "You have to ask whoever is in charge how it works," and "I don't know; can you tell me once more?".

## Discussion

### Effectiveness

The results from this study showed that familiarity may have a substantial role in reducing errors, as the odds of a participant committing an error more-or-less match faucet familiarity ordering for all three groups (as presented in Figure 1 and Table 4). Interestingly, participants from the moderate group committed more errors on the dual lever, crosshead, and infrared than the severe group (as presented in Figures 2a and 3). In part, this is a result of the higher levels of instruction and support given to the severe group by the RA (as presented in Figure 4); a large proportion of the severe groups’ trials had hand-over-hand assistance or were completed by the RA, either of which would result in errorless step completion. Another factor may have been that people with moderate dementia were on the cusp of requiring assistance; they were cognisant enough to understand that they were being asked to turn on the water and felt that this was something they should be able to do, however, they were unable to remember how to do so, which resulted in many attempts and errors.

It appears that the types of errors that were made may be more closely related to the faucet design than to dementia level. For instance, for all groups location errors were the most common error type for the infrared while operation errors were high for the plastic wand (as presented in Figure 3). This supports the notion that a faucet’s design provides intuitive cues about how to operate it and lack thereof can impede use. As the same faucet designs tended to cause the same type of error for all groups, it seems that implicit cues are perceived similarly for people with dementia as they are by people without a cognitive impairment, albeit at a greater rate. Interestingly, the odds of committing an error on the most familiar design, the crosshead, were not statistically different from the less familiar infrared, and in the case of the moderate group, the plastic wand (as presented in Figure 3 and Table 4). In part, this is because the rotational operation of the faucets handles caused outcome errors for the crosshead (i.e., participants knew to turn the handle, but turned it in the wrong direction), decreasing the crosshead’s overall odds of errorless use.

### Efficiency

Unsurprisingly, these results indicate the need for assistance increases considerably as cognitive abilities decrease (as presented in Figures 2b and 4). Overall, the caregiver assistance data support the supposition that familiarity plays a significant role in product use for people with dementia, with more familiar faucets resulting in less assistance. Moreover, familiarity may well have a greater impact on required assistance as cognitive abilities decrease, demonstrated by the aware/mild group using all faucets more-or-less independently compared to the severe group, who required the least assistance for the familiar dual lever, crosshead, and single lever designs, noticeably more assistance for the less-familiar (but more “usable”) infrared, and assistance for every trial with the unfamiliar plastic wand.

### Satisfaction

Two observations regarding the self-reported difficulty ratings (as presented in Figure 5) are especially interesting. The first is that, with the exception of the plastic wand, self-reported perceived difficulty is similar across all faucet designs for the same group. The second is that perceived difficulty seems to increase with dementia severity. This suggests that perceived difficulty is more dependent on the respondent’s cognitive abilities than on the faucet type. Although it cannot be determined definitively from this study, it is plausible that as dementia progresses and cognitive impairments become more severe, people are aware that using a faucet becomes more difficult, regardless of the design. This theory is supported by comments made by participants, which reflect increasing trouble with faucet use as dementia progresses.

When it is considered with the other data from this study, self-reported difficulty captured by the Likert question appears to reflect both perceived (participants’ comments) and empirically measured (errors and assistance) levels of difficulty. As such, the verbally administered Likert question seems to have been successful. To the authors’ knowledge, this is the first work to employ a Likert-based approach to capture people with dementias’ perceptions regarding product use. These results suggest that the verbal administration of a single, simple Likert question in stages is a viable and promising method of eliciting self-perceived difficulty of product use by older adults with dementia, particularly for those at the mild to moderate stages. These results mirror research to ascertain pain and quality of life, such as [40]. However, as gauging the reliability of using a Likert question with people with dementia to obtain perceived difficulty was not within the scope of this study, this needs to be definitively addressed through future research.

### Overall faucet use

The dual lever faucet appears to achieve the best overall usability, resulting comparable or lower odds of a person requiring assistance or making an error (Table 2) for all groups. Self-perceived difficulty (Figure 5) with the dual lever appears to be comparable or better than other faucets and participants’ comments reflected acceptance of the dual lever, such as “You just need a finger to use it,” and “I would have this in my home.” It is important to note that the participants used dual lever faucets in their rooms at the long-term care facility where this study was conducted. While the dual lever used in this research was a different model than the one in the participants’ rooms, daily exposure to a dual lever design could have had a non-trivial priming effect on the faucet familiarity and may have impacted study results.

There are three lines of reasoning that can explain the dual lever’s better usability: 1) there was greater lifetime exposure to the dual lever than other faucet types; 2) the definition of familiarity for older adults with dementia and its resulting impact on product use should include significant recent exposures, and; 3) the dual lever design is easier for older adults to use. Likely, it is a combination of these three propositions that resulted in the dual lever’s good usability; a lifetime of experience, recent exposure (i.e., dual lever faucets were in the participants’ rooms), and a design that is easy for people with morbidities to use that also provides implicit feedback (i.e., the dual lever’s actuators’ positions indicate flow rate and temperature).

It appears that familiarity does significantly impact the odds of an older adult being able to use a faucet, particularly if he or she is at the moderate stage of dementia. This stipulation is supported by people in all groups using the infrared faucet (a design considered to be more usable from a human factors perspective but less familiar) with less success than the more familiar crosshead, dual lever, and single lever designs. While trends in assistance and errors suggest that familiarity plays a greater role in supporting product use as dementia worsens, this cannot be stated definitively as all groups tended to have more success with more familiar faucet designs. Looking at the amount and type of assistance required (as presented in Figures 2b and 4 and Table 4), many people from the severe group may be at a stage of dementia where they are no longer able to complete the task of turning on the water independently, regardless of the type of faucet that is used. As such, perhaps it is worth focusing on usability for people at the aware, mild, and moderate stages when selecting a faucet (or other product) design. Satisfaction was higher for more familiar designs, although this may have been substantially influenced by the control over temperature and flow that these designs offer.

Ensuring individuals are able to control over water flow and temperature could result in increased feelings of satisfaction, which could encourage autonomous initiation of self-care tasks, promote engagement in the task, and increase feelings of dignity and control. This research indicates that for washroom tasks, the plastic wand and infrared faucets are not the first choice for older adults, both with and without dementia. Empirical (as presented in Figures 2, 3, and 4 and Table 4) lends support for this preference from measured effectiveness and efficiency perspectives. Thus while issues such as water usage and the ease of cleaning are important (particularly in a health care facility), it is essential that these factors are carefully weighed against product usability, user preferences, and product acceptance.

### Study limitations

There are several limitations that should be considered when interpreting the results of this research. All participants were residents of the same long-term care facility, therefore samples from other facilities and communities could produce different results. Additionally, comorbidities are more prevalent in long-term care residents than in community-dwelling older adults, which may have exacerbated difficulties with faucet use. Exposure to the faucets within the long-term care facility could have priming effects, particularly the use of dual-lever faucets in the participants’ private washrooms at the residence. It is important to bear in mind that participants were under observation by researchers, therefore most attempted to use each faucet, even if he or she disliked a particular model. Ergo, while most participants were able to use all the faucets, some may have chosen to not use a faucet they disliked in an unsupervised setting. Finally, this work only investigated faucet use during the task of handwashing, therefore findings may not be transferable to other products or tasks.

## Conclusion

While no one faucet design truly stood out, the dual lever achieved better performance than the other designs and was accepted by older adults, both with and without dementia, as a design they recognised and liked. The supposition that product familiarity has a greater impact on how usable a product is than the product’s usability (as defined by a human factors approach) for people with dementia appears to be supported by this research. In the majority of cases, the more classic crosshead and dual lever designs appeared to elicit better performance than the more “usable” single lever and infrared designs. This research also supports the notion that people with dementia are able to express opinions regarding product design and use. Moreover, the methods employed in this research could be used to include people with dementia in the development of products, which could enable designers to incorporate preferences and abilities to ensure new products are more intuitive to and usable by a wider range of people; both with and without dementia.

There are many interesting results that would benefit from further investigation, such as the progression of self-perceived difficulty as dementia level increases. Long-term studies to understand how ADLs are impacted by the familiarity and usability of other products would provide a great deal of insight into how older adults’ preferences and attitudes towards design impacts independence. The methods described in this work could be adapted for the analysis of use of any product by people with dementia; the measures presented here for gauging effectiveness, efficiency, and satisfaction could be used in high-tech or low-tech product use analysis. However, as these methods are resource intensive, conducting a large-scale study would be a significant undertaking. To address this, work is underway to develop computer algorithms that can autonomously capture and categorise product use, allowing large amounts of data to be analysed automatically with the goal of enabling a more holistic understanding of product use [46,47].

The research presented in this paper indicates that familiarity plays a substantial role in faucet usability for older adults with dementia that becomes more influential as dementia progresses. This research also shows that while different designs may result in similar levels of assistance and errors, where and how the faucet will be used should be carefully considered when selecting a design. While it must be remembered that this study examined only the activity of handwashing in a washroom environment (and the water on step in particular), these results shed considerable light on how older adults, both with and without dementia, perceive and use faucets. While these trends may well be applicable to other products and activities, future research is required to extend these conjectures with certainty.

## Abbreviations

ADL: Activities of daily living; RA: Research assistant (Caregiver).

## Competing interests

The authors declare that they have no competing interests.

## Authors’ contributions

JB participated in writing the study proposal and grant, wrote and submitted ethics materials, coordinated study execution, assisted in running trials, assembled data, performed the majority of data analysis and wrote and revised the majority of the manuscript. TC performed recruitment, acted as the caregiver for the trials, performed video annotations, assisted with data analysis, and assisted with manuscript preparation. AM participated in writing of the study protocol and grant, assisted in data analysis, and assisted in manuscript preparation. All authors read and approved the final manuscript.

## Pre-publication history

The pre-publication history for this paper can be accessed here:

http://www.biomedcentral.com/1471-2318/13/63/prepub
